# Promising Results of an Exclusive Radiotherapy Treatment of an Inoperable Giant Eyelid Sebaceous Carcinoma

**DOI:** 10.7759/cureus.61592

**Published:** 2024-06-03

**Authors:** Asmae Hamdan, Salma El Baz, Hanan El Kacemi, Tayeb Kebdani, Khalid Hassouni

**Affiliations:** 1 Department of Radiotherapy, National Institute of Oncology, Mohammed V University, Faculty of Medicine, Rabat, MAR

**Keywords:** case report, giant, inoperable, exclusive radiotherapy, sebaceous carcinoma

## Abstract

Eyelid sebaceous carcinoma is a rare malignant tumor. Surgical excision is generally the standard curative treatment. However, in cases where surgery is not possible due to locally advanced tumors, definitive radiotherapy can be considered an option. A 71-year-old man presented with a history of eyelid sebaceous carcinoma dating back two years. The tumor, measuring 93 x 55 x 56 mm and located on the right upper eyelid, was not surgically resected. He was then treated with intensity-modulated radiotherapy, receiving a total dose of 70 Gy in 35 fractions. After 24 months, the patient shows no local recurrence.

## Introduction

Ocular sebaceous carcinomas originate from the meibomian glands (tarsal), Zeis glands (eyelash), or sebaceous glands of the eyelid and caruncle [1]. Eyelid sebaceous carcinoma is a rare tumor, accounting for less than 1% of all eyelid tumors and about 5% of all eyelid malignancies [2,3]. The median age of affected patients is between 70 and 73 years [4,5]. The upper eyelid is most often involved [4].

Histologically, the tumors are composed of large cells with abundant clear or basophilic cytoplasm with a foamy appearance [6]. Lymphatic spread is the most frequently observed, but distant metastases can also be present, mainly involving the liver, lungs, and bones [7]. The risk of metastasis is closely related to a size greater than 10 mm and to extensive invasion, as represented by tumors classified as T3-4 [8]. The diagnosis of eyelid sebaceous carcinoma can be delayed because it can mimic other inflammatory conditions, such as chalazion, stye, blepharitis, keratoconjunctivitis, or other lesions. Indeed, the average time between the onset of symptoms and diagnosis is about two years [2,9].

Surgery is generally recommended as the treatment of choice for this tumor, which was previously considered resistant to radiotherapy [5]. However, some studies have suggested that radiotherapy might also be effective [10]. We report a case of eyelid sebaceous carcinoma that completely responded after exclusive radiotherapy.

## Case presentation

**Figure 1:**
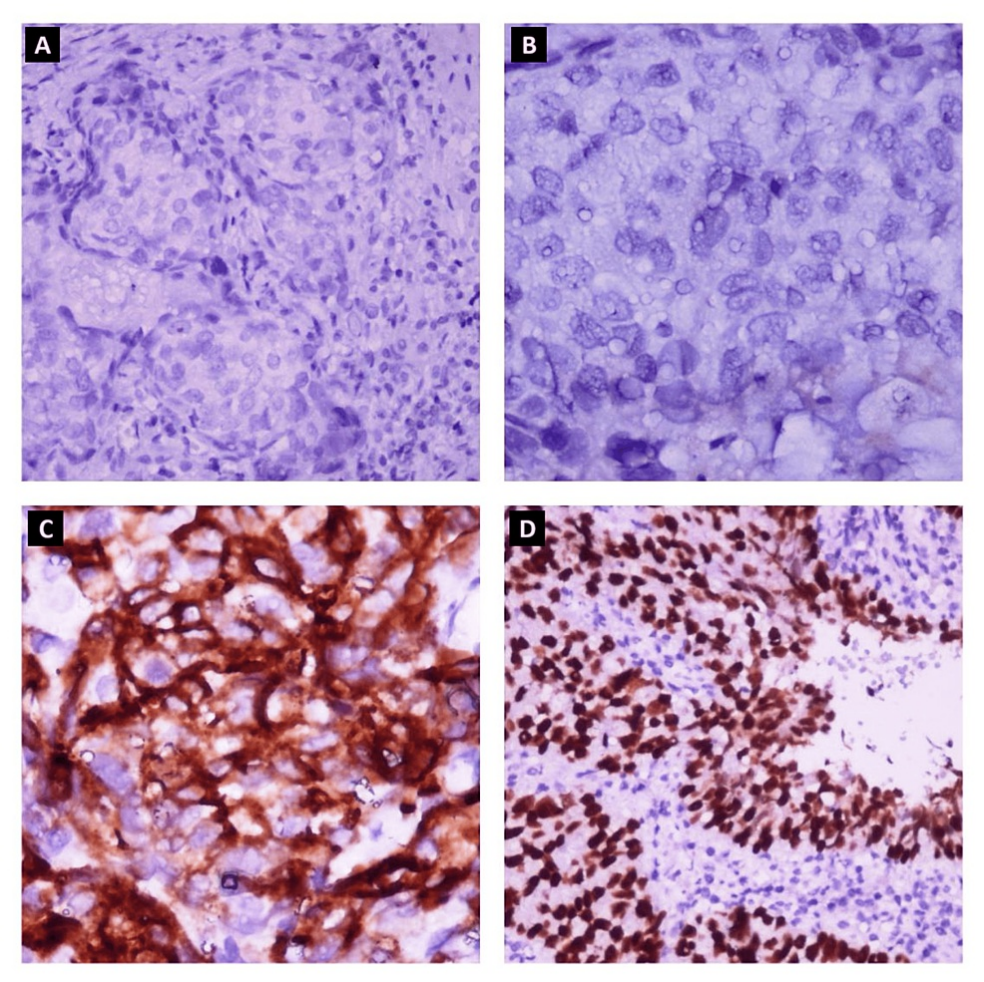
Immunohistochemical staining. A: Absence of staining of tumor cells for Melan A (IHC x20). B: Absence of staining of tumor cells for PS100 (IHC x40). C: Partial positive staining of tumor cells for EMA (IHC x200). D: Diffuse positive staining of tumor cells for P40 (IHC x400). Melan A: marker Melan-A, PS100: protein S-100, EMA: epithelial membrane antigen, P40: marker specific to the p40 subunit of the p63 protein

A 71-year-old Moroccan man (North Africa) with no significant personal or family medical history. The history of this disease dates back two years with the appearance of a painless nodule on the right upper eyelid, which gradually increased in size without any other associated signs. Surgical intervention was recommended, but the patient refused and was lost to follow-up for one year, until the lesion became a giant bleeding mass. The pathological examination of a biopsy of the lesion revealed moderately differentiated sebaceous carcinoma. Immunohistochemical staining results were positive for EMA, CEA, P40, and P63 but negative for PS100 and Melan A (Figure 1). Computed tomography of the orbit and brain revealed a right orbital process with frontal sinus and intracranial extension, accompanied by homolateral lymphadenopathies. An MRI orbit-cerebral was performed, describing the right orbital process centered at the level of the eyelid soft tissue, with dimensions of 93 x 55 x 56 mm. The disease was classified as T4bN2bM0 from the eighth edition of the American Joint Committee on Cancer staging form supplement.

**Figure 2:**
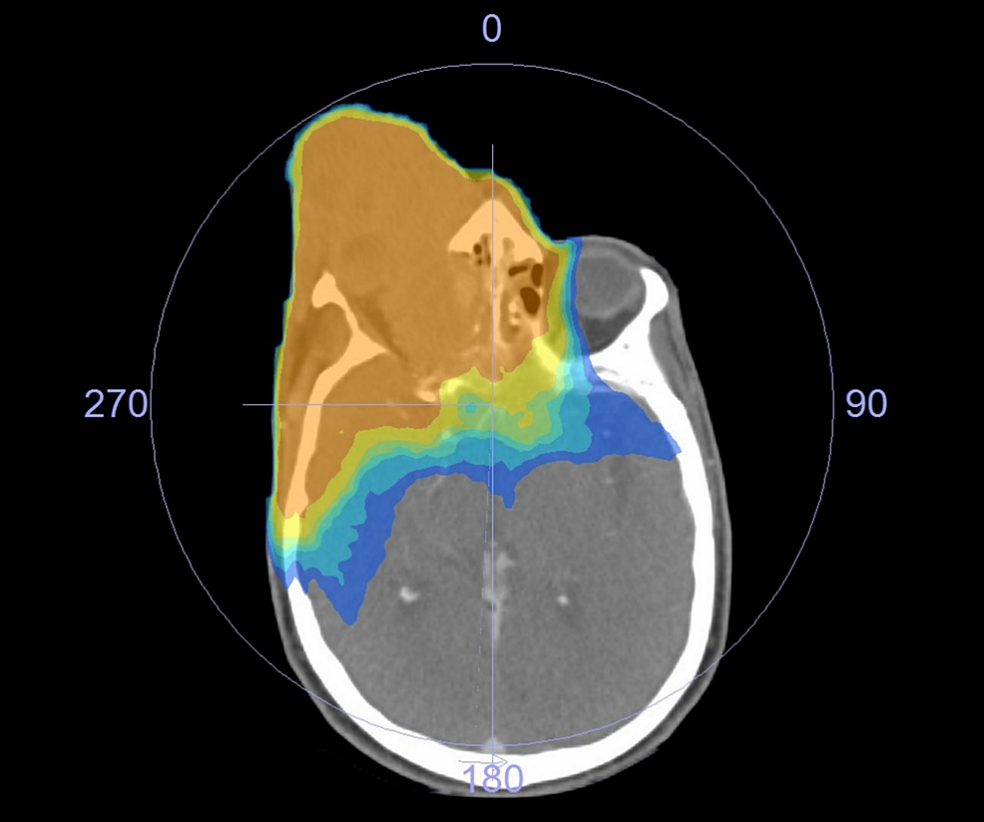
Radiotherapy isodose curve (axial view): the planning CT scan with 60 Gy (in yellow) and 70 Gy (in orange) isodose lines.

**Figure 3:**
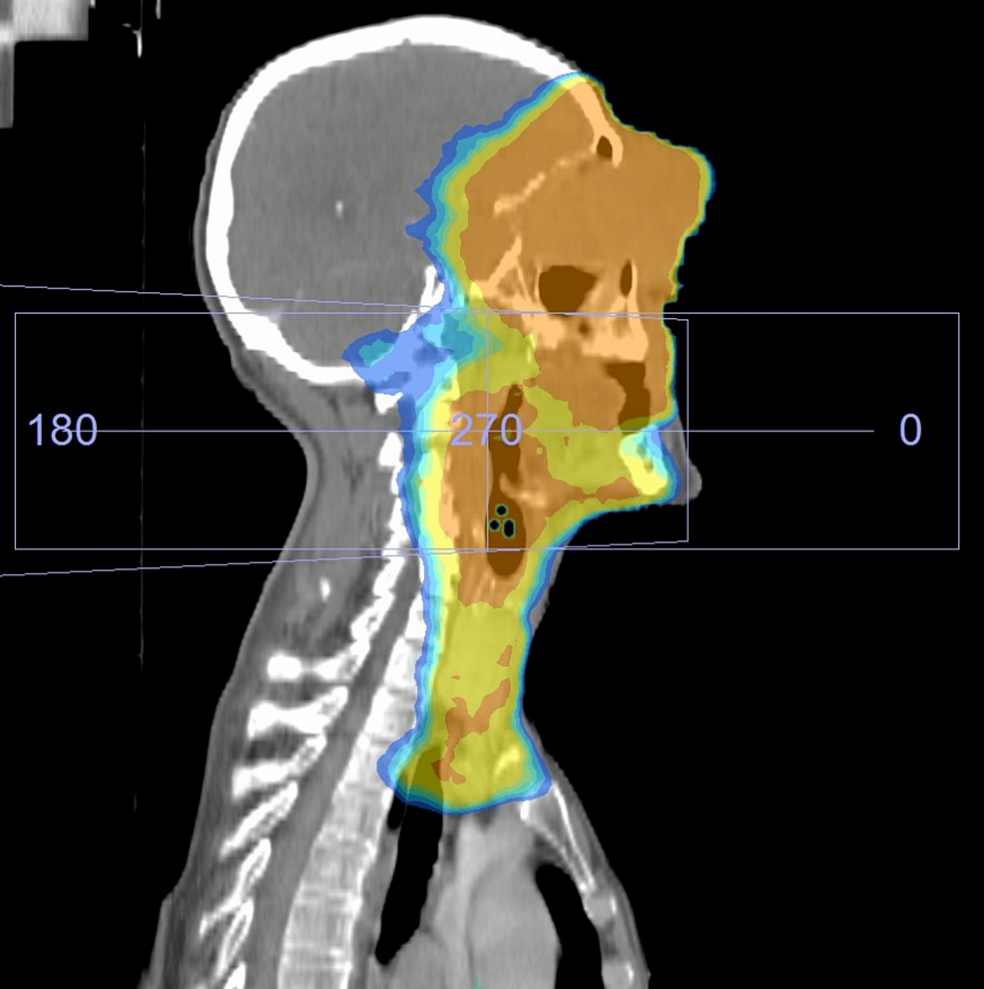
Radiotherapy isodose curve (sagittal view): the planning CT scan with 60 Gy (in yellow) and 70 Gy (in orange) isodose lines.

Due to the very advanced local progression of the tumor, vision deterioration, difficulty in opening the eyelid, and bleeding on contact, surgery was no longer considered. Definitive radiotherapy was administered after discussion with the patient, with a dose of 60 Gy in 30 fractions on the tumor and the nodal areas and a boost of 10 Gy in five fractions on the tumor and the lymph nodes (Figures 2, 3).

The treatment plan was established using intensity-modulated radiotherapy (IMRT). The simulation was performed using the CT simulator. The patient was immobilized in a supine position with a five-point thermoplastic head and neck mask. Planning CT images were acquired with intravenous iodine contrast and a slice thickness of 3 mm. The CT DICOM images were transferred to the treatment planning system for the delineation of target volumes. The acquired images were then co-registered for the delineation of target volumes, including the gross tumor volume (GTV), the clinical target volume (CTV), and the planning target volume (PTV). The gross target volume (GTV) includes the primary tumor and gross nodal disease at initial presentation. The CTV1 includes the GTV with a 5 mm margin and the ipsilateral neck levels IB, II, III, IV, and V since the lymphadenopathy was at levels IB, II, III, and IV. The CTV2 includes the GTV and a 3 mm margin. The PTV1 and PTV2 are defined as the CTV1 and CTV2 with a 5 mm and 3 mm margin, respectively. A prescription dose of 60 Gy in 30 fractions was given to PTV1, with an additional 10 Gy in five fractions to PTV2. Organs at risk (OARs) were contoured according to the Radiation Therapy Oncology Group (RTOG) atlas for normal tissue contouring. Volumetric modulated arc therapy (VMAT) with a two-arc technique plan was generated using 6 MV photon beams. PTV coverage and dose to OARs were acceptable. Dose constraints to OARs were defined according to the RECORD. The VMAT plan was delivered with Versa HD (Elekta, Sweden). Patient setup was verified daily by cone beam CT imaging before treatment.

The patient developed grade II radiodermatitis according to the Common Terminology Criteria for Adverse Events (CTCAE) version 4.0, but the limitation of eye-opening and the deterioration of visual acuity were present before the start of treatment.

**Figure 4:**
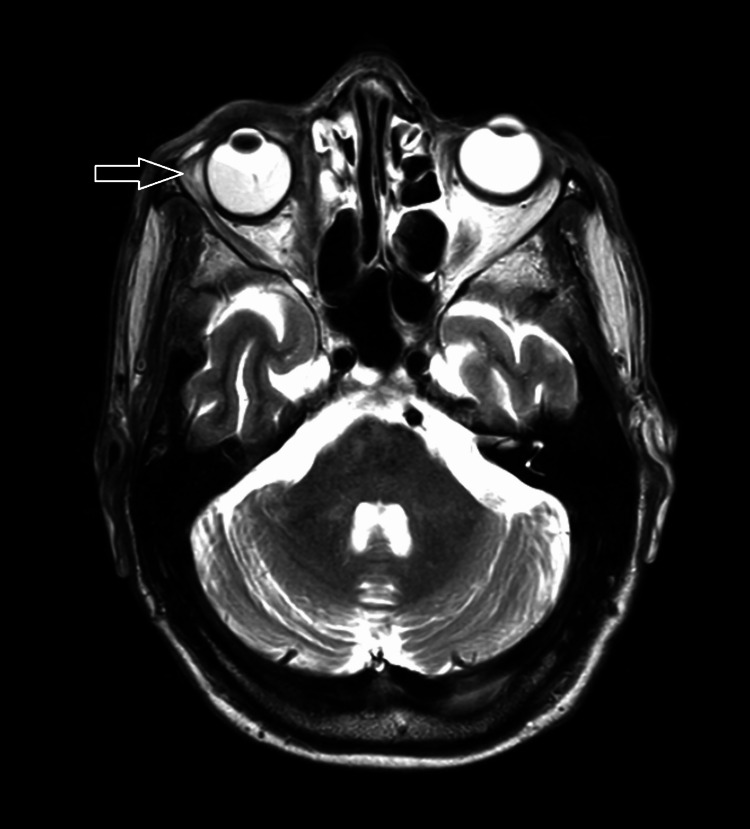
MRI taken 3 months after radiotherapy (T2-weighted image from the axial plane showing a decrease in the size of the right orbital process with intermediate signal).

Three months after the treatment, an MRI showed a 48% partial response, measuring 14 x 25 x 27 mm compared to 93 x 55 x 56 mm initially (Figure 4). After 24 months, the patient did not show any clinical lesions, and there was no recurrence. Regarding side effects, no ulceration was present, but the patient had limited eye-opening.

## Discussion

Eyelid sebaceous carcinoma is highly malignant and potentially aggressive [11]. Surgical excision has long been considered the only curative treatment, as this lesion was traditionally seen as radio-resistant [12]. Therefore, there is little data on the effectiveness of radiotherapy in treating this condition. Despite recent advances in reconstructive surgery, complete excision of eyelid tumors can be very difficult without functional or aesthetic impairment. Radiotherapy may be an option for cases where surgery is not possible or when patients refuse surgical intervention, but its role in the treatment of eyelid sebaceous carcinoma remains uncertain. It is possible that the total doses of radiotherapy administered to tumors in the past were insufficient [13,14].

In recent years, numerous studies and case reports have shown promising results of radiotherapy in the treatment of locally advanced diseases, demonstrating its effectiveness as an alternative to surgery and its potential for achieving local control [14-16]. A study involving 83 patients with eyelid sebaceous carcinoma showed that radiotherapy produces comparable results for tumors smaller than or equal to 10 mm and promising results in patients at stages T3 and T4 [17]. The optimal dose for curative radiotherapy of eyelid sebaceous carcinoma remains unknown [12], but doses higher than 55 Gy seem to be associated with better local control [18]. Others recommend a minimum dose of 60 Gy in conventional fractions to eradicate the gross tumor [14,15]. In general, doses ranging from 56 to 70 Gy administered in 2 Gy fractions can be considered [13].

Regarding the results, the study of 83 patients revealed that radiotherapy achieved a local control rate of 46.6% over seven years, even for larger eyelid sebaceous carcinomas (>10 mm, including T4) [17]. Another major study on radiotherapy as monotherapy included 78 patients with eyelid sebaceous, with an average disease-free survival of 54% at five years [19]. This disease can sometimes lead to cervical lymph node metastases, particularly metastases to the preauricular, parotid, or submandibular lymph nodes, observed in 8% to 32% of patients [9,20]. Prophylactic irradiation could be effective in preventing cervical lymph node metastases and seems necessary for patients with T3-4 tumors [17]. Regarding the toxicities associated with radiotherapy for eyelid tumors, in addition to acute reactions, such as dermatitis and conjunctivitis/keratitis, the development of cataracts has been reported [15].

The results of this case will reinforce the evidence in favor of primary radiotherapy for eyelid sebaceous. In this case, it was not necessary to limit the dose to preserve visual acuity. The treatment was deemed appropriate and effective, despite the presence of numerous unfavorable prognostic factors.

## Conclusions

While surgical excision was traditionally considered the only curative treatment for an eyelid sebaceous carcinoma due to its reputation for radiotherapy resistance, our study has demonstrated that radiotherapy can be a promising curative option in place of surgery for the treatment of an eyelid sebaceous carcinoma.

Furthermore, the literature supports that radiotherapy has proven to be effective and safe in treating this pathology, ensuring good tumor control, and offering a perspective on cure. Although the optimal dose of radiation remains to be determined, a total dose of at least 60 Gy in conventional fractions might be necessary to achieve complete control of advanced tumors.
